# Conservative Management of a Complicated Crown Fracture Using Autogenous Fragment Reattachment and Fiber Reinforcement: A Case Report

**DOI:** 10.7759/cureus.117218

**Published:** 2026-09-30

**Authors:** Nithin S Reddy, Ashwija Shetty, Hajira A Sultana, Ashritha M A, Varsha K

**Affiliations:** 1 Department of Conservative Dentistry and Endodontics, The Oxford Dental College, Bengaluru, IND

**Keywords:** complicated crown fracture, dental trauma, fiber-reinforced composite, fragment reattachment, partial pulpotomy

## Abstract

Complicated crown fractures involving pulpal exposure present both biological and restorative challenges, particularly when preservation of pulp vitality and natural tooth structure is desired. Autogenous tooth fragment reattachment is a conservative treatment option that preserves the original morphology, contour, surface texture, and optical characteristics of the fractured tooth. However, the bonded interface may remain mechanically vulnerable to functional loading; therefore, contouring and internal groove preparation are recommended to improve fracture strength recovery. This case report describes the conservative management of a complicated crown fracture in a 23-year-old male patient using partial pulpotomy with mineral trioxide aggregate (MTA), followed by autogenous tooth fragment reattachment with preheated resin composite, over-contouring, and fiber reinforcement. The patient presented approximately three hours after an accidental fall with a complicated crown fracture of the maxillary left central incisor involving enamel and dentin with pulp exposure. The tooth exhibited a positive response to cold sensibility testing, consistent with a responsive pulp, and showed no tenderness to percussion or mobility. Following partial pulpotomy and placement of MTA over the remaining pulp tissue, the fractured fragment was reattached using preheated resin composite. An over-contouring approach was subsequently performed on the buccal surface, and Interlig fiber (Angelus, Londrina, Brazil) was incorporated into a prepared groove for additional reinforcement. At the one-year follow-up, the tooth remained asymptomatic, responded positively to cold sensibility testing, and showed no tenderness to percussion, fragment loss, discoloration, periodontal ligament widening, or periapical pathology. This case illustrates a conservative approach combining vital pulp therapy, autogenous fragment reattachment, and localized fiber reinforcement while preserving natural tooth structure and esthetics.

## Introduction

Traumatic injuries to anterior teeth frequently result in loss of coronal tooth structure. Complicated crown fractures, characterized by pulpal exposure, present an additional biological challenge because treatment must address both the structural defect and the exposed pulp. When the fractured fragment is available and demonstrates satisfactory adaptation to the remaining tooth, autogenous fragment reattachment is a conservative restorative option. Reattachment preserves the original tooth anatomy, contour, surface texture, translucency, and optical characteristics while minimizing the removal of sound tooth structure [1,2].

Preservation of pulpal health is an important consideration in the management of complicated crown fractures. Partial pulpotomy permits the removal of the superficially inflamed pulp tissue while preserving the remaining vital pulp. Favorable outcomes have been reported following vital pulp therapy in traumatized permanent teeth with complicated crown or crown-root fractures, although the quality of available clinical evidence remains variable [3,4]. Mineral trioxide aggregate (MTA) is widely used as a pulp-protective material due to its favorable biological properties and ability to provide a suitable barrier over the remaining pulp tissue. However, traditional MTA carries a risk of discoloring anterior teeth. To address this, newer white MTA formulations were introduced to prevent discoloration. Furthermore, the successful management of traumatized permanent incisors using partial pulpotomy with MTA has been reported in conjunction with the adhesive reattachment of fractured tooth fragments [5].

Although adhesive reattachment is highly conservative, the reattached tooth may remain mechanically weaker than an intact tooth. Various preparation techniques, including beveling, chamfering, internal groove preparation, and over-contouring, have therefore been investigated to improve the fracture resistance of reattached teeth [1,2,6,7]. Over-contouring increases the amount of composite surrounding the fracture region and may provide additional reinforcement while preserving the original tooth fragment [6,7]. However, these findings are laboratory-based and may not directly translate to superior long-term clinical performance.

The restorative material used for reattachment may also influence adaptation to the bonding surfaces. Preheating reduces the viscosity and increases the flowability of composite resins, allowing for better adaptation to cavity walls, particularly with high-viscosity materials. By minimizing microleakage, this technique enhances restoration durability. Furthermore, the elevated thermal energy boosts free radicals and monomer mobility, which accelerates the polymerization rate and leads to a higher degree of monomer conversion [8,9]. Fiber reinforcement has also been investigated as an adjunct to fragment reattachment, with laboratory studies reporting potential improvements in fracture resistance; however, clinical evidence remains limited [10-12].

The present case describes the conservative management of a complicated crown fracture using partial pulpotomy and MTA followed by autogenous fragment reattachment with preheated resin composite. An over-contouring approach with localized fiber reinforcement was subsequently employed to provide additional reinforcement while preserving natural tooth structure and esthetics.

## Case presentation

A 23-year-old male patient reported to the Department of Conservative Dentistry and Endodontics with a fractured maxillary anterior tooth following an accidental fall approximately three hours before presentation. Clinical examination revealed a complicated crown fracture of the maxillary left central incisor involving enamel and dentin with pulp exposure (Figure 1A-1B). The patient was asymptomatic, and the fractured fragment was intact with satisfactory adaptation to the remaining tooth structure (Figure 1C). The patient had preserved the fragment intraorally in the buccal vestibule following the traumatic incident. The fragment was disinfected with 2% chlorhexidine solution and was stored in saline for 15 minutes before reattachment.

**Figure 1:**
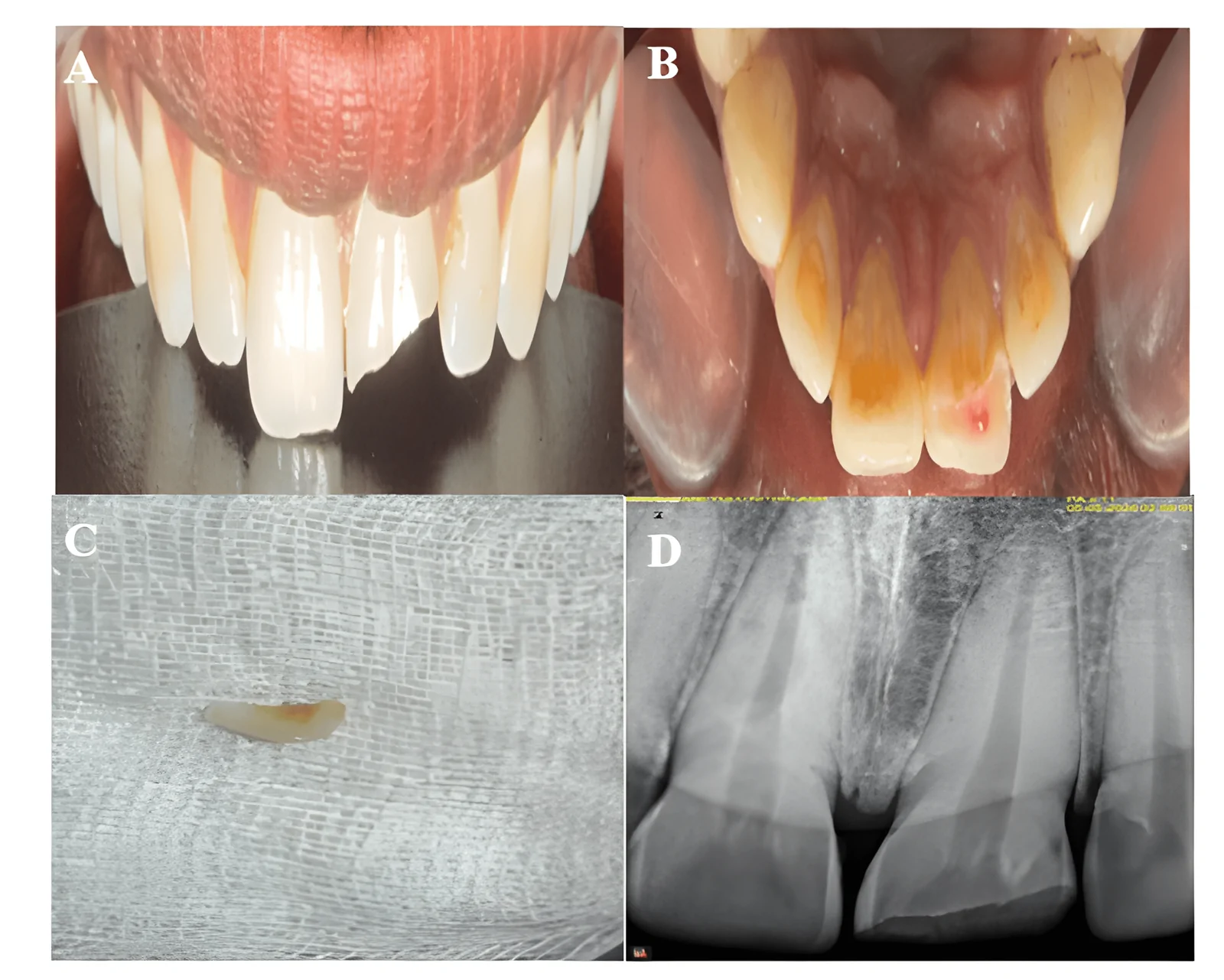
(A) Preoperative clinical image, (B) preoperative clinical image showing the crown fracture with pulpal involvement, (C) fractured tooth fragment, and (D) preoperative radiograph

The tooth showed no tenderness to percussion. Cold sensibility testing elicited an exaggerated response that subsided upon the removal of the stimulus, compared with the contralateral tooth. Preoperative radiographic examination demonstrated a fracture involving the enamel and dentin with pulpal exposure, without evidence of periodontal ligament widening, periapical pathology, or any associated root fracture (Figure 1D). Based on the clinical and radiographic findings, a diagnosis of complicated crown fracture associated with reversible pulpitis was established.

The shade was selected prior to rubber dam isolation. Following the administration of local anesthesia by local infiltration using 2% lignocaine and epinephrine (1:80,000), the tooth was isolated with a rubber dam. Partial pulpotomy was performed under a dental operating microscope to remove approximately 2 mm of inflamed coronal pulp tissue (Figure 2A). The exposed pulp tissue was gently irrigated with saline, and a sterile cotton pellet soaked in 2.5% sodium hypochlorite solution was placed to achieve hemostasis. After approximately 5-10 minutes, the cotton pellet was removed, revealing healthy, bright-red pulp tissue with adequate control of bleeding. Subsequently, approximately 1.5 mm of MTA (Angelus, Londrina, Brazil) was placed over the remaining pulp tissue as a protective barrier (Figure 2B). A postoperative radiograph was obtained to verify the position and adaptation of the MTA (Figure 2C). Glass ionomer cement (Shofu, Kyoto, Japan) was subsequently placed over the MTA barrier.

**Figure 2:**
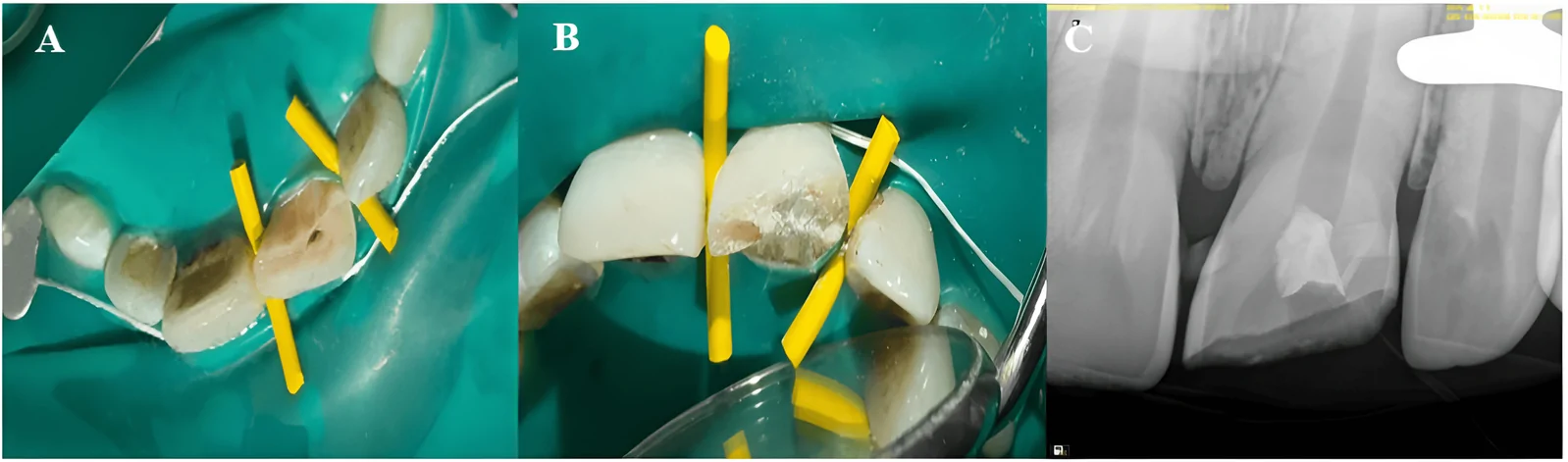
(A) Clinical image following partial pulpotomy, (B) mineral trioxide aggregate placement following partial pulpotomy, and (C) radiographic confirmation of mineral trioxide aggregate placement

Both the tooth and fragment surfaces were etched with 37% phosphoric acid gel for 30 seconds, followed by thorough rinsing. The bonding adhesive was then applied to the etched surfaces and light-cured for 20 seconds. Packable composite resin (Filtek™ Z250 XT Nano Hybrid Universal Restorative, 3M, Maplewood, Minnesota, United States) was preheated to 54.5°C for 15 minutes using a composite warmer (EndoKing Nanowarm, Vasa Denticity Pvt. Ltd., New Delhi, India) and applied to both the tooth and fragment surfaces. The fractured fragment was carefully repositioned onto the tooth, ensuring accurate adaptation and intimate contact between the fractured segments. After confirming proper alignment, excess composite resin was removed, and the assembly was light-cured for 20 seconds. Following fragment reattachment, an over-contouring approach was performed on the buccal surface. A groove approximately 1 mm in depth and 0.8 mm in width was prepared, extending 1 mm into the tooth surface and 1 mm into the fractured fragment (Figure 3A-3C). Following etching and the application of the bonding agent to the prepared surface, pre-impregnated Interlig fiber (Angelus, Londrina, Brazil), measuring approximately 2 mm in length, was incorporated into the prepared groove and covered with preheated resin composite (Figure 3D-3E), followed by light curing. The restoration was subsequently finished and polished to reproduce the original tooth contour, surface texture, occlusion, and esthetics (Figure 3F).

**Figure 3:**
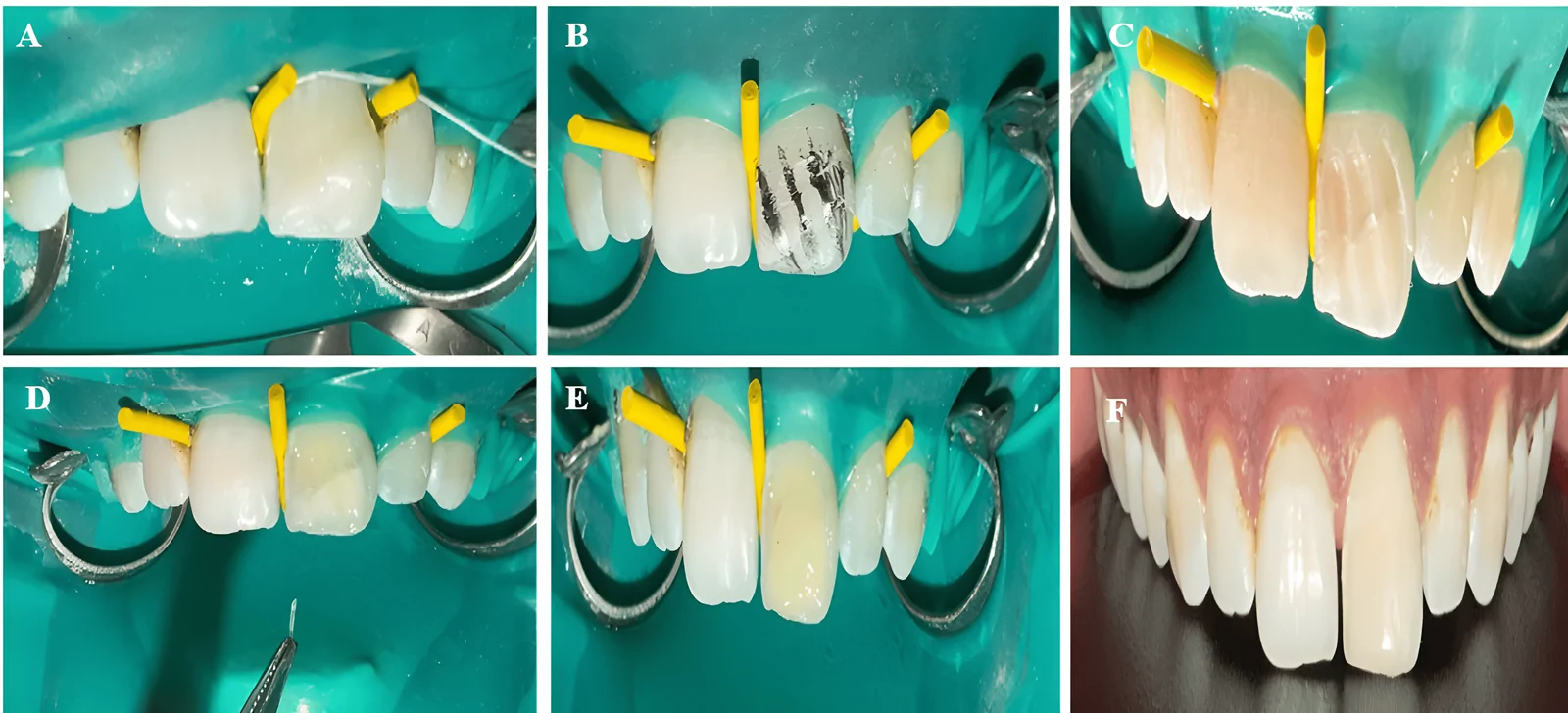
(A) Initial repositioning and reattachment of the fractured tooth fragment, (B) marking of the area for buccal over-contouring, (C) preparation of the buccal surface according to the planned over-contouring design, (D) placement of Interlig fiber within the prepared surface, (E) application of preheated resin composite for fiber coverage and the restoration of the buccal contour, and (F) clinical image following finishing and polishing

The patient was recalled at one week and at one, three, six, and 12 months. At each follow-up examination, the tooth remained asymptomatic and showed no tenderness to percussion or clinically evident tooth discoloration and no evidence of fragment loss (Figure 4A-4B). Cold sensibility testing demonstrated a normal response compared to the contralateral tooth. Follow-up radiographs demonstrated no periodontal ligament widening or periapical pathology (Figure 4C-4D). The clinical and radiographic findings remained favorable throughout the one-year follow-up period.

**Figure 4:**
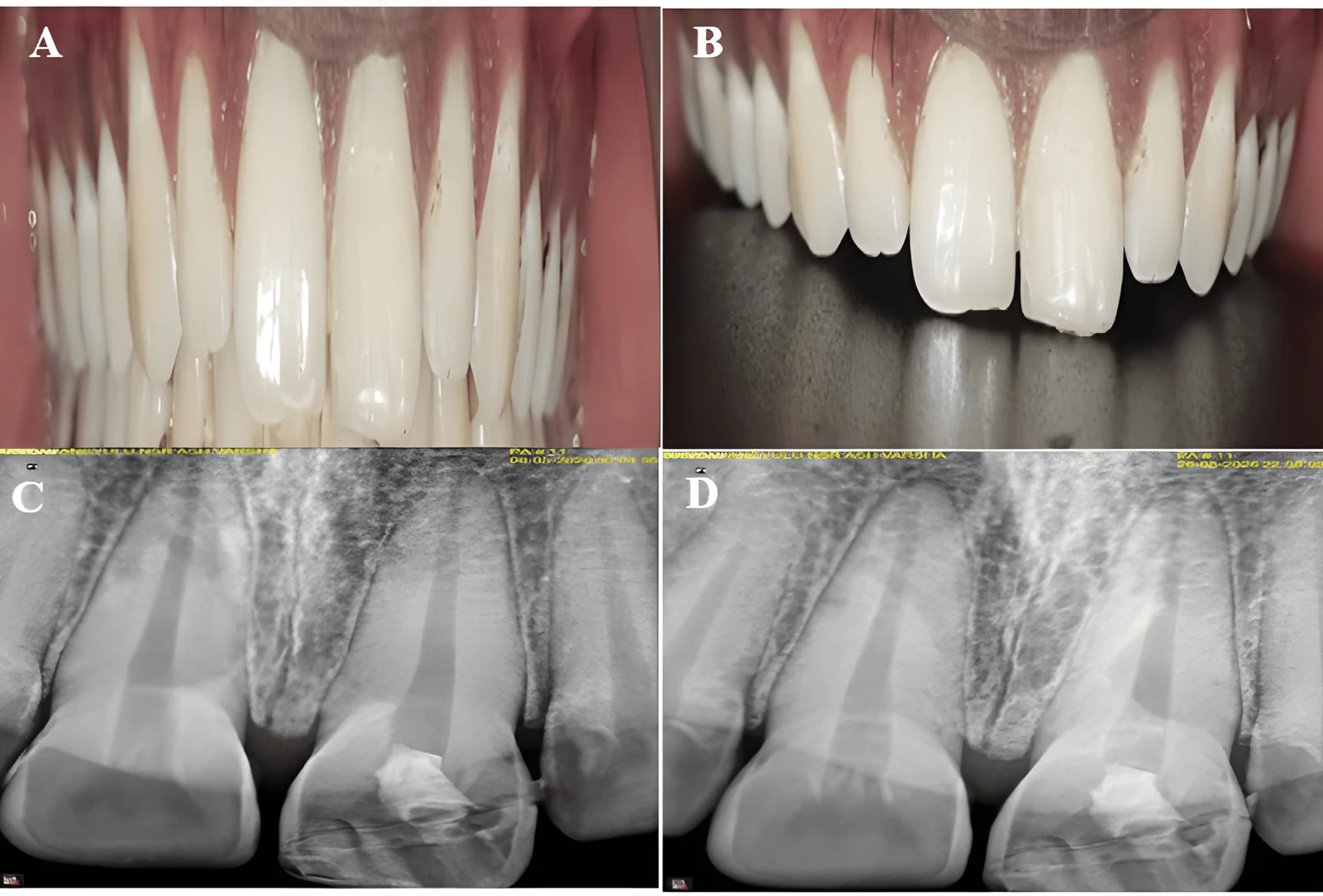
(A) Clinical appearance at the six-month follow-up, (B) clinical appearance at the 12-month follow-up, (C) radiographic appearance at the six-month follow-up, and (D) radiographic appearance at the 12-month follow-up

## Discussion

Complicated crown fractures involving pulpal exposure present both restorative and biological challenges. Treatment should aim to preserve the remaining tooth structure and maintain pulp vitality whenever predictable vital pulp therapy is feasible [3,4]. In the present case, the availability of an intact coronal fragment with satisfactory adaptation allowed restoration using the patient's own tooth structure rather than replacing the lost anatomy with an artificial restoration. Fragment reattachment is considered a minimally invasive approach because it preserves the original morphology, surface texture, contour, and optical characteristics of the natural tooth [1,2].

Although simple fragment reattachment is conservative when adequate adaptation is achieved, the bonded interface may remain mechanically vulnerable to functional loading. Garcia et al. [1] reported considerable variation in fragment reattachment techniques and highlighted the lack of consensus regarding a universally superior technique. Similarly, a systematic review of in vitro reattachment techniques demonstrated substantial variation in preparation designs and restorative approaches [2]. Experimental studies have shown that preparation techniques such as over-contouring and internal groove preparation can improve fracture strength recovery compared with simple reattachment [6,7]. Therefore, the additional reinforcement used in the present case was intended to provide localized mechanical reinforcement rather than to imply superiority over other reattachment techniques.

The pulpal exposure in the present case was managed using partial pulpotomy rather than immediate root canal treatment, with the objective of preserving the remaining vital pulp tissue. Donnelly et al. [3] reported favorable outcomes for pulpotomy in permanent teeth with complicated crown fractures, although the authors emphasized limitations in the available evidence, including methodological limitations and small sample sizes. Matoug-Elwerfelli et al. [4] similarly reported favorable outcomes for vital pulp treatment in traumatized permanent teeth, with partial pulpotomy being the most frequently reported intervention. These findings support partial pulpotomy as a conservative treatment option when the clinical conditions are favorable.

MTA was placed over the remaining pulp tissue because of its favorable biological properties and ability to provide an effective barrier over the pulp [5]. Previous reports have demonstrated successful outcomes with MTA-assisted partial pulpotomy combined with adhesive reattachment of autogenous tooth fragments in traumatized mature permanent teeth, with favorable follow-up findings ranging from 12 months to two years [5,10,11]. These findings support the combined use of vital pulp therapy and adhesive fragment reattachment when preservation of pulp vitality is clinically feasible. However, traditional gray MTA may pose a risk of tooth discoloration; therefore, white MTA was used in the present case to minimize the potential for discoloration.

The present case incorporated an over-contouring approach to reinforce the fracture region following fragment reattachment. Several preparation designs, including beveling, internal grooves, and over-contouring, have been investigated to improve the fracture resistance of reattached teeth [6,7]. Pusman et al. [6] demonstrated differences in fracture resistance according to preparation technique and adhesive material, while Reis et al. [7] reported differences in fracture strength according to the reattachment technique employed. Thus, over-contouring in the present case should be considered a conservative reinforcement strategy rather than an assertion that it is superior to all other preparation designs.

Preheated composite was used during fragment reattachment to facilitate manipulation and adaptation of the restorative material. Preheating decreases the viscosity of resin composite and can facilitate its adaptation to bonding surfaces [8]. Elkaffas et al. [9] reported a beneficial effect of composite preheating on surface hardness in a systematic review and meta-analysis. However, the effects of preheating vary according to the composite material and temperature protocol, and available evidence does not establish that preheating consistently improves all mechanical properties. Therefore, in the present case, the primary rationale for preheating was improved handling and adaptation of the composite at the tooth-fragment interface rather than an assumption of guaranteed enhancement of fracture resistance.

Additional fiber reinforcement was incorporated into the prepared groove using Interlig fiber and subsequently covered with composite. The rationale for this approach was to provide localized reinforcement across the reattachment region and potentially distribute functional stresses over a broader area. A systematic review by Albar identified seven in vitro studies evaluating fiber-reinforced approaches for reattached tooth fragments. Although several studies demonstrated increased fracture resistance following fiber reinforcement, the review concluded that the overall evidence supporting improved fracture resistance was of low quality [12].

Fragment loss remains a recognized complication of adhesive reattachment. Tewari et al. [13] reported that bonded fragment loss represents a clinically relevant outcome following fragment reattachment, although the certainty of the available evidence was very low. Therefore, additional reinforcement may be considered in selected cases, but the current evidence does not establish that fiber reinforcement prevents future fragment loss. In the present case, the retained fragment at the one-year follow-up represents a favorable clinical outcome but cannot by itself establish the long-term protective effect of the fiber-reinforced approach.

At the one-year follow-up, the tooth remained clinically asymptomatic, responded positively to cold sensibility testing, and demonstrated no fracture, mobility, or discoloration. Satisfactory occlusion was maintained, and radiographic examination revealed dentin bridge formation without evidence of periapical pathology. The favorable pulpal outcome is consistent with reported outcomes following partial pulpotomy for complicated crown fractures [3,4]. However, the follow-up of a single case cannot establish long-term treatment success or determine the relative contribution of individual treatment components.

The principal strength of the present approach was the integration of biological preservation and conservative restorative rehabilitation within a single treatment sequence. The natural coronal fragment was preserved, the remaining pulp tissue was maintained through partial pulpotomy, and localized mechanical reinforcement was provided using over-contouring and fiber placement [3,5,10,11]. This approach may be particularly relevant in anterior teeth, where preservation of natural morphology and optical characteristics is important in addition to biological and mechanical considerations [1,2].

Nevertheless, this report has inherent limitations associated with its single-case design and absence of a comparative control. The individual contributions of partial pulpotomy, MTA, over-contouring, preheated composite, and fiber reinforcement cannot be isolated from the clinical outcome. Furthermore, much of the evidence supporting preparation techniques, preheated composite, and fiber reinforcement is derived from laboratory studies, limiting direct extrapolation to long-term clinical performance [8,12]. Longer follow-up and larger clinical studies are required to determine the durability of fragment retention and the contribution of individual reinforcement techniques.

## Conclusions

The present case describes a conservative approach to managing a complicated crown fracture when an intact, well-adapted tooth fragment is available. Partial pulpotomy was performed to preserve the remaining pulp tissue, while autogenous fragment reattachment restored the original coronal anatomy and esthetic characteristics. Over-contouring and localized fiber reinforcement were employed to reinforce the reattachment interface, while preheated composite facilitated material adaptation. The favorable clinical and radiographic findings at the one-year follow-up are encouraging. However, as this is a single case without a comparative control, the long-term effectiveness and individual contribution of each treatment component cannot be established. Longer follow-up is required to evaluate sustained pulpal health and fragment retention.
